# *Solanum jobsonii*, a novel andromonoecious bush tomato species from a new Australian national park

**DOI:** 10.3897/phytokeys.82.12106

**Published:** 2017-06-28

**Authors:** L. Mae Lacey, Jason T. Cantley, Christopher T. Martine

**Affiliations:** 1 Department of Biology, Bucknell University, 1 Dent Drive, Lewisburg, PA, USA

**Keywords:** Limmen National Park, Northern Territory, *Solanum watneyi*, *Solanum eburneum*, *Solanum diversiflorum*, *Solanum jobsonii*, Peter Jobson, andromonoecy, national parks, public lands, Australia

## Abstract

A new species of *Solanum* from the Australian “andromonoecious bush tomato clade” of Solanum subgenus Leptostemonum is described. *Solanum jobsonii* Martine, J.Cantley, & L.M.Lacey, **sp. nov.** is part of the *S. eburneum* Symon species group. It most closely resembles *S. eburneum* and *S. watneyi* Martine & Frawley of the northwestern part of the Northern Territory, but is separated geographically from them by the Sturt Plateau. Morphometric analyses show that *S. jobsonii* differs statistically from *S. eburneum*, *S. watneyi*, and *S. diversiflorum* F.Muell. – a similar species in habit and leaf characters – in several key reproductive and vegetative characters. We provide morphometric evidence for the recognition of *S. jobsonii*, a complete description, a table of comparisons within its species group, and a map showing species group distributions. One of the first new species to be described from Limmen National Park (established 2012), *S. jobsonii* is a testament to the value of designating and protecting public lands, as well as supporting science relating to them.

## Introduction

*Solanum* L. is one of the more species-rich Angiosperm genera, with representation on all continents save for Antarctica. In Australia, where upwards of 120 *Solanum* species are known (Symon 1981), members of the group are especially abundant components of disturbance-adapted and fire-tolerant outback plant communities; and a handful of species have been used for thousands of years by indigenous peoples as “bush tucker” (Peterson 1976, Symon 1981, Doonday et al. 2013).

However, despite the conspicuous nature of the genus in parts of Australia, new species of *Solanum* have continued to be discovered in recent years – especially members of the “spiny solanum” group (Solanum subgenus Leptostemonum Bitter) in the northern Australian Monsoon Tropics (Brennan et al. 2006, Bean and Albrecht 2008, Barrett 2013, Martine et al. 2013, Bean 2016, Martine et al. 2016a, Martine et al. 2016b).

The “andromonoecious bush tomato clade” is a group of 12 currently recognized species that was recognized by Martine and colleagues based on analysis of ITS (Martine et al. 2006) and trn-KmatK sequence data (Martine et al. 2009). Included in the clade are two morphologically similar species groups, one comprised of *S. chippendalei* Symon, *S. succosum* A.R. Bean & Albr., *S. beaugleholei* Symon, and *S. phlomoides* A.Cunn. ex Benth., and another comprised of *S. eburneum* Symon, *S. watneyi* Martine & Frawley and *S. diversiflorum* F.Muell. Unnamed variants are known to exist within each of these groups that require the collection of more specimens. One of these variants, from the *S. eburneum* group, is described here as *Solanum jobsonii* sp. nov.

Reproductive populations of *Solanum jobsonii* piqued the curiosity of Australian botanists during 2008 and 2010 biodiversity surveys (see Cowie et al. 2011) of the proposed Limmen National Park on the edge of the Gulf of Carpentaria in northeastern Northern Territory. Collections of these plants, initially identified as S. aff. eburneum, were brought to the attention of the authors by the staff of the Northern Territory Herbarium and inspired a collecting expedition to the recently-designated Limmen NP in 2016. Specimens from this expedition and seed-grown greenhouse plants were used to conduct morphometric comparisons between *S. jobsonii* and the three previously recognized taxa in the *S. eburneum* group. We here contrast both its morphology and distribution with its close relatives, and provide a table of comparisons for members of the *S. eburneum* group.

## Materials and methods

Based on locality data provided on specimens available at DNA and BUPL herbaria (acronyms according to Index Herbariorum; http://sweetgum.nybg.org/science/ih/), the primary known populations of *S. jobsonii* were visited along the Nathan River Road in Limmen NP. Herbarium specimens, leaf material for future DNA work, and mature fruits were collected. Seeds of the putative new species were removed from fresh fruits, dried, and stored for later use in establishing a greenhouse population.

Plants were grown for use in ex situ morphometric analyses by soaking field-collected seeds in 1000-ppm gibberellic acid for 24 hours, then sowing them in a controlled growth chamber environment at Bucknell University (Pennsylvania, USA). Seeds germinated in 2-3 weeks and plants were cultured under Integrated Pest Management conditions. Twenty-six vegetative and reproductive characters were measured across developmental stages. Leaf lobe depths were based on measures from the base of the most deeply cut sinus to the tip of the nearest lobe. All morphological data were then compared against related species collected during the 2016 expedition and specimens examined during visits to the Northern Territory Herbaria at Palmerston (DNA) and Alice Springs (NT).

Comparison statistics were generated using the software package JMP Pro 12 (SAS Institute Inc., Cary, North Carolina, USA). Initial analyses were carried out on the dataset using a one-way ANOVA with Student’s t-test mean comparison with P<0.05. A Connecting Letters Report summarizes mean values of each character across the four study taxa (*S. watneyi*, *S. eburneum*, *S. diversiflorum*, *S. jobsonii*) and provided similarity comparisons based on calculated means and tests carried out. This Connecting Letters Report was utilized to investigate individual differences between species before analyzing grouping differences through multivariate morphometric analyses.

Multivariate morphometric analyses were then conducted on the entire dataset for all four species. Principal component analysis (PCA) was used to determine morphological variation pattern groupings among the four taxa. A set of variables was plotted based on corresponding eigenvalues calculated using JMP Pro, representing the original dataset with the greatest variation in a two-dimensional space.

## Results

**Figure 1.**
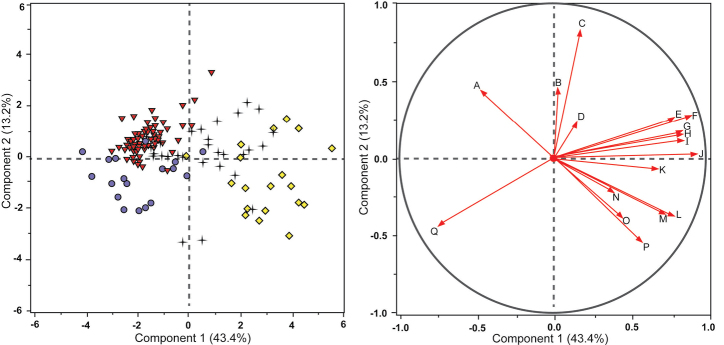
Principal components analysis score plot (Fig. 1a, left) and loading plot (Fig. 1b, right) of characters and species in Table 1. Most heavily weighted characters labeled and indicated with red arrows in loading plot. Red triangles = *S. jobsonii*; black crosses = *S. eburneum*; purple circles = *S. diversiflorum*; yellow diamonds = *S. watneyi*. Labels for loading plot are as follows: **A** Apical leaf depth of lobing **B** Basal leaf width **C** Basal leaf depth of lobing **D** Stem prickle length **E** Hermaphrodite calyx lobe length **F** Staminate calyx lobe length **G** Basal leaf length **H** Male corolla diameter **I** Hermaphrodite corolla diameter **J** Apical leaf length **K** Apical leaf surface area **L** Petiole length **M** Internode length **N** Apical leaf width **O** Plant height **P** Fruiting pedicel length **Q** Seed length.

The PCA recognized five eigenvalues above a value of 1 and these were used to determine that the data set was in its entirety five-dimensional, with principal components 1 and 2 contributing the greatest amount of variation among the points (56.6% of the variation within the data set). Figure 1 provides both the score plot with each of the data points plotted, as well as the loading plot. The loading plot depicts which characters had the greatest weight in pulling out the points within the score plot to each of their respective quadrants, therefore determining which characters had the greatest influence in delimiting the species. When reading Figure 1, one may mentally superimpose the two plots to view the points and the corresponding vectors whose magnitude represents the associated character’s weight in plotting the dataset and grouping of individuals, but as the loading plot is much smaller it is represented as a separate panel to depict species character influence described above.

The PCA score plot based on all measured characters for *S. watneyi*, *S. eburneum*, *S. diversiflorum*, and *S. jobsonii* supports the relative distinctiveness of *Solanum jobsonii* based on character grouping when compared to the other three closely related taxa, although some overlapping with *S. diversiflorum* and *S. eburneum* variants is observed (Figure 1a). The loading plot (Figure 1b) suggests that the depth of lobing in young leaves near the apices of growing shoots (identified in Table 1 as “apical leaves”) was the character with the greatest weight pulling *S. jobsonii* in the direction of its respective quadrant.

These results, in conjunction with ANOVA comparisons of each individual character across the four taxa support the hypothesis that *S. jobsonii* is a distinct entity. The addition of student’s t-tests also provided a Connecting Letters Report (Table 1). The table is to be read horizontally across each of the four taxa as a means to compare across discrete characters. Taxa maintaining separate letter distinctions for any one measured character are recognized as being significantly different from the other three taxa in that character. Nine characters (highlighted in Table 1) meet this criterion for *Solanum jobsonii*, including corolla diameter (in both staminate and hermaphrodite flowers), calyx lobe length, and length of the fruiting pedicel.

Table 2 presents an additional set of non-numerical characters that one can use for drawing distinctions between the four taxa of the *S. eburneum* species group (Table 2).

**Table 1.** Vegetative, floral, and fruiting characters measured for *S. watneyi*, *S. eburneum*, *S. diversiflorum*, and *S. jobsonii* with associated mean (M), standard deviation (SD), sample size (n), and Connecting Letters Report (CL) which indicates distinctions among species by character. Species not connected by the same letter in a row are significantly different (p < 0.05) for that character. All measurements in cm, except seeds per fruit, seed length and fruit wall width (mm), leaf surface area (cm^2^), and trichome density (per 0.5 cm^2^ area). “Apical” refers to leaves near the tips of growing stems, while “Basal” refers to leaves borne on lower portions of the stems. The nine characters found to be significantly different in *S. jobsonii* when compared to the other three taxa are in bold.

Character	*S. watneyi*				*S. eburneum*				*S. diversiflorum*				*S. jobsonii*			
	M	SD	n	CL	M	SD	n	CL	M	SD	n	CL	M	SD	n	CL
Stem prickle length	0,26	0,26	25	BC	0,40	0	16	A	0,22	0,09	30	C	0,29	0,12	54	B
**Internode length**	4,01	0,98	25	A	2,16	0,67	16	B	2,23	0,91	30	B	1,48	0,51	54	C
Petiole length	3,36	3,36	25	A	2,68	0,80	16	B	1,03	0,53	30	C	0,82	0,39	54	C
**Apical leaf length**	12,39	2,50	25	A	11,32	0,10	16	A	2,66	0,77	25	C	5,13	1,39	35	B
Apical leaf width	2,47	0,65	25	A	1,41	0,41	16	B	1,61	0,41	25	B	1,48	1,17	35	B
**Basal leaf length**	16,80	3,85	25	A	13,66	1,83	16	B	5,80	1,98	25	D	9,18	1,87	29	C
**Basal leaf width**	3,97	1,15	25	B	2,03	0,88	16	D	3,24	0,91	25	C	4,94	1,62	29	A
Apical leaf adaxial trichome density	382,60	98,36	5	A	243,40	88,79	5	B	215,60	90,21	5	B	316,20	60,91	5	AB
Apical leaf abaxial trichome density	518,20	147,30	5	A	331,00	137,05	5	B	420,60	116,48	5	AB	491,60	119,88	5	AB
Basal leaf adaxial trichome density	--	--	--	--	--	--	--	--	28,40	3,21	5	--	42,80	10,21	5	--
Basal leaf abaxial trichome density	--	--	--	--	--	--	--	--	116,20	36,92	5	--	108,40	33,91	5	--
Apical leaf depth of lobing	0,27	0,36	10	C	0,61	0,36	20	B	0,79	0,24	15	AB	0,90	0,62	15	A
Basal leaf depth of lobing	--	--	--	--	--	--	--	--	1,48	0,38	15	--	2,01	0,92	15	--
Apical leaf surface area	10,67	6,54	25	A	3,49	2,54	25	B	2,54	1,17	25	B	3,02	1,93	25	B
Basal leaf surface area	--	--	--	--	--	--	--	--	9,99	4,12	25	--	19,86	7,68	25	--
**Staminate corolla diameter**	4,01	0,62	25	A	3,46	0,31	16	B	2,24	0,45	16	D	3,02	0,44	23	C
**Hermaphrodite corolla diameter**	4,75	0,58	25	A	4,03	0,35	16	B	3,00	0,37	16	D	3,58	0,50	17	C
**Staminate calyx lobe length**	1,13	0,14	7	A	--	--	--	--	0,35	0,09	15	C	0,88	0,14	10	B
**Hermaphrodite calyx lobe length**	2,23	--	1	A	--	--	--	--	0,35	0,09	6	C	1,16	0,14	14	B
**Fruiting pedicel length**	4,22	0,65	19	A	3,45	0,64	14	B	2,70	0,18	13	C	1,65	0,34	12	D
Fruit length	2,15	0,34	29	B	1,80	0,29	13	C	3,11	0,29	14	A	1,65	0,13	3	C
Fruit width	1,96	0,36	29	BC	2,20	0,41	13	B	2,92	0,35	14	A	1,68	0,28	3	C
Seeds per fruit	53,11	28,45	28	C	78,69	36,67	13	B	433,00	--	1	A	101,67	58,60	3	B
Plant height	45,85	6,90	25	A	43,62	10,86	16	AB	33,80	4,44	3	BC	34,50	6,26	7	C
Seed length	3,05	0,18	20	B	2,84	0,21	20	C	4,11	0,25	15	A	3,09	0,21	15	B
Fruit wall width	5,50	--	1		3,10	--	1		4,40	--	1		2,20	--	1	

**Table 2.** Comparison of key qualitative characters across the four closely-related taxa of the *Solanum eburneum* species group, including *S. jobsonii*.

Character	*S. watneyi*	*S. eburneum*	*S. diversiflorum*	*S. jobsonii*
Plant habit	sprawling/lax, open	erect, compact	lax branches and leaves	erect or lax, compact
Lobing of leaves, # lobes	± shallow (if present), few, 0–2 per leaf	deep, numerous, 6–8 per leaf	pinnatifid, 4–8 per leaf	dissected, 6–12 per leaf
Corolla color	lighter purple, ‘dusty purple’	darker purple, ‘mauve’	medium purple	medium purple
Corolla margins	wavy, undulating	more or less flat	repand	involute
Fruit shape	± ellipsoidal	± globose	± globose	± globose
Fruit color at maturity	yellow, ‘light lemon’ with light brown striping	white, ‘creamy’ without striping	light yellow-green with dark green striping	yellow, creamy yellow
Fruit interior at maturity	more or less dry	liquid-filled	liquid-filled	liquid-filled
Fruit firmness at maturity	firm	soft, squishy	firm	firm
Fruit position at maturity	pendant, on or near ground	pendant from stems, but not on ground	pendant from stems, but not on ground	pendant from stems, but not on ground
Seed color	light to dark brown	black	tan to light brown	dark brown to black
Soil type	well-drained limestone based sandy- or clayey-loamy soil	gray clay soil	red sandy soil	clayey soil with laterite pebbles

### Taxonomic treatment

#### Solanum jobsonii

Taxon classificationAnimaliaSolanalesSolanaceae

Martine, J.Cantley, & L.M.Lacey sp. nov.

urn:lsid:ipni.org:names:77163784-1

Fig. 2

##### Diagnosis.

With affinity to *Solanum eburneum*, *Solanum watneyi*, and *Solanum diversiflorum*, but differing by the involute corolla margins, deeply dissected leaves with 6–12 lobes and smaller creamy-yellow fruits.

##### Type.

AUSTRALIA. The Northern Territory: Limmen National Park, on main road, 15°54'47"S, 135°31'43"E, elev. ca. 250 ft, 12 May 2010 (fl, fr), *B. Stuckey & I.D. Cowie 645* (holotype [two sheets]: DNA)

##### Description.

Lax to weakly erect sub-shrub or short-lived perennial herb to 20–50 cm tall. Stems slender, woody, upright even when weighted by fruits; initially single stemmed, with strong lateral branching beginning at ca. 7 cm; internode length on mature stems ca. 1.5 cm. Overall plant aspect dark green to gray-green, becoming slightly more yellow-green with age; pubescence of stems short and loose; moderately to densely pubescent throughout with stellate stalked trichomes, the stalk 0.05–0.1 (rarely to 0.2) mm long, with 6–8 rays 0.2–0.4 mm long, the midpoint elongate, to 0.4 mm long. Prickles sparse to moderately dense, straw-colored, straight, slightly widened at base, fine, 1–6 mm long, scattered on stems. Sympodial units difoliate, the leaves solitary or geminate. Mature leaves 5–9 cm × 1.5–5 cm, linear to lanceolate or elliptic, with 1–4 pairs of primary veins, with only a few prickles along midvein on leaf undersides; young leaves lighter green and gray-hairy but becoming dark green above, slightly paler beneath, both sides closely and densely stellate-pubescent, the older leaves becoming scabrous and uniformly dark on both sides, retaining dense pubescence primarily only along veins; base tapering; margins deeply incised and 6–12 lobed, occasionally shallowly lobed or nearly linear; apex blunt; petiole 0.3–2.3 cm long with few to no prickles. Inflorescence a supra-axillary andromonoecious cyme 1–6.5 cm long, consisting of a basal hermaphrodite flower and a distal group of 2–5 (usually) staminate flowers; typically 2–5 staminate flowers open at a time; common peduncle typically 1.5–2.5 mm long; rachis slightly less pubescent than stems. Flowers 5-merous, heterostylous with a single hermaphroditic flower at the base of the inflorescence and the plants andromonoecious. Hermaphrodite flower ca. 1.5–3 cm below the staminate flowers, usually opening first or soon after lowest 1–2 staminate flowers; pedicel ca. 2 cm long at anthesis, elongating further after fertilization, armed with prickles 1–4 mm long; calyx lobes ca. 11–15 mm long and fused for first 2–3 mm, some pairs occasionally fused entirely with sepals arranged 2+2+1, armed with long, straight prickles and stellate trichomes; corolla 2.5–4.5 cm in diameter, medium purple, rotate, free of indumentum; stamens equal; filaments ca. 1.5 mm long; anthers 5 mm long, oblong-lanceolate to somewhat tapered, poricidal at the tips, in a tight anther cone; ovary glabrous, ca. 2 mm diameter at anthesis; style 6–11.5 mm long (including capitate stigma), curved. Staminate flowers with pedicels 9–14 mm long, unarmed or with few prickles; calyx lobes 6–10 mm long and fused for first 1–2 mm, occasionally 2+2+1 as above, with a few 1–4 mm weak prickles or prickles absent; corolla 2.5–3.5 cm in diameter, medium purple, broadly stellate to rotate; acumens ca. 0.5 mm long; stamens of same proportions as in hermaphrodite flower; ovary, style, and stigma vestigial and not exserted beyond the stamens. Fruit a globose berry 1.6–1.8 cm long, 1.5–2.0 cm wide, light green with darker green stripes when young, maturing to creamy yellow; flesh firm; locules 2, liquid-filled; fruit wall ca. 2.2 mm thick; fruits retained on plant after maturation. Fruiting pedicels 1.2–2.3 cm long. Fruiting calyx enclosing and exceeding fruit in early development, eventually covering 1/4 to 1/3 of developed fruit, the lobes narrowly triangular, long-acuminate, blunt-tipped, turning brown and weakly reflexing as fruit matures, short stellate-pubescent and armed with sharp spines 2–5 mm long, these single or paired along the calyx sutures. Seeds up to ~135 per fruit, 2.8–3.6 mm long, dark brown to black, flat, reniform, finely reticulate.

**Figure 2.**
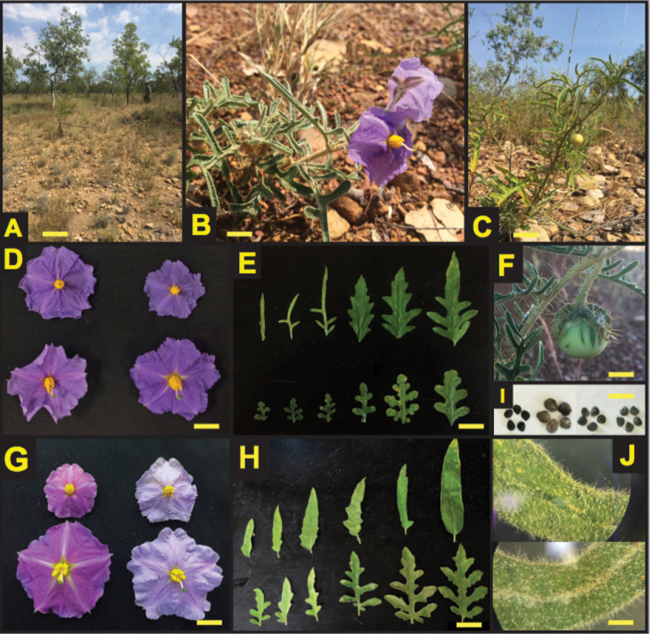
*Solanum jobsonii* Martine, J.Cantley, and L.M.Lacey and related species. **A** Typical habitat in clay soils with limestone stones and laterite pebbles, Limmen National Park, NT **B** *S. jobsonii* in flower and **C** in mature fruit **D** Corolla comparisons of staminate (upper) and hermaphrodite (lower) flowers for *S. jobsonii* (left) and *S. diversiflorum* (right) **E** Leaf shape across varying leaf ages for *S. jobsonii* (top) and *S. diversiflorum* (bottom) **F** *S. jobsonii* immature fruit with armed calyx **G** Corolla comparisons of staminate (upper) and hermaphrodite (lower) flowers for *S. eburneum* (left) and *S. watneyi* (right) **H** Leaf shape across varying leaf ages for *S. watneyi* (top) and *S. eburneum* (bottom) **I** Seed size, shape, and color comparisons from left to right – *S. jobsonii*, *S. diversiflorum*, *S. eburneum*, and *S. watneyi* **J** *S. jobsonii* trichome density of apical adaxial leaf surface (top) and apical abaxial leaf surface (bottom). Photos **A, B, C, F, G** by J.T. Cantley; **H** by E.S. Frawley; **D, E, I, J** by L.M. Lacey. Yellow scale bars: **B, D, F, G** = 1.5 cm; **C** = 5 cm; **E, H** = 2.25 cm; **I** = 8 mm; **J** = 1.5 mm.

##### Distribution and ecology.

**Figure 3.**
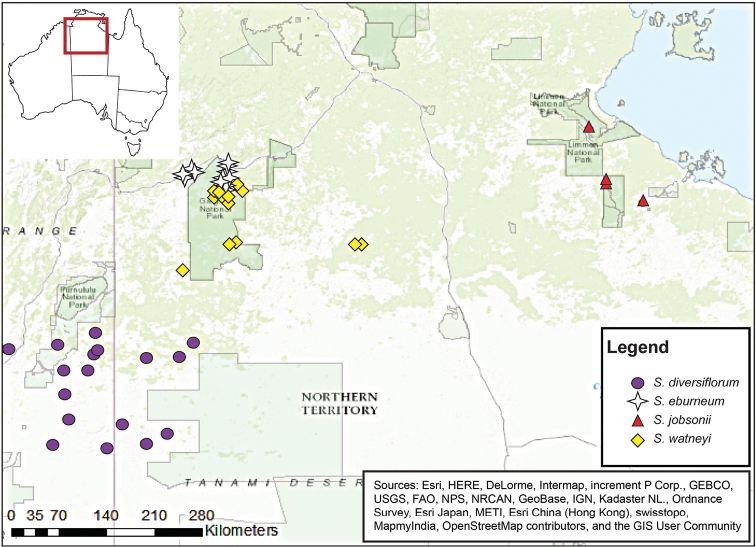
Geographic distribution of *S. watneyi*, *S. eburneum*, *S. diversiflorum*, and *S. jobsonii* in eastern Western Australia and the Northern Territory, Australia, based on specimens held at the Northern Territory Herbarium Palmerston (DNA).

*Solanum jobsonii* is presently known mostly from a restricted range of localities in Limmen NP in the sub-arid, monsoon-influenced zone of northeastern Northern Territory (Fig. 3) at elevations around 250 feet. The species is locally abundant in a few sites along and just off of the Nathan River Road, yet abundance elsewhere is not known. *Solanum jobsonii* is primarily associated with *Eucalyptus pruinosa* Low Open Woodland (Cowie et al. 2011) on seasonally-flooded alluvial plain fringes (above seasonal streams) and plains. The most abundant population encountered in 2016 was in a Eucalyptus pruinosa Schauer subsp. pruinosa (Myrtaceae) woodland on grey-brown clay with limestone stones and laterite pebbles where the primary associated taxa were *Melaleuca nervosa* (Lindl.) Cheel (Myrtaceae), *Dodonaea physocarpa* F.Muell. (Sapindaceae), *Dolichodandrone heterophylla* (R.Br.) F. Muell. (Bignoniaceae), *Grewia retusifolia* Kurz (Malvaceae), *Carissa lanceolata* R.Br. (Apocynaceae), *Eulalia aurea* (Bory) Kunth (Poaceae), *Calandrinia gracilis* Benth. (Portulacaceae), herbs and grasses. Although *S. jobsonii* is nearly always found in *E. pruinosa* woodlands, the converse is not true; *S. jobsonii* was not present in many of the *E. pruinosa* stands in which we searched. This suggests that *S. jobsonii* is sensitive to fine-scale habitat variation that we did not observe.

Nothing is known about pollination biology or seed dispersal of *S. jobsonii*, but floral morphology aligns with the typical *Solanum* buzz pollination syndrome (see Anderson and Symon 1988) and the fleshy berries suggest biotic dispersal (see Symon 1979). Plants encountered in May 2016 bore numerous mature fruits that had not been taken by frugivores – a phenomenon also seemingly typical among close relatives.

Although *S. jobsonii* has been collected on the edges of graded roads and appears to be disturbance-adapted, the species only appears where these thoroughfares bisect otherwise suitable habitat where seasonal flooding is also apparent. Occasional bushfires figure prominently into the ecology of these sites, but the effect on *S. jobsonii* is unknown.

##### Phenology.

The few collections made of flowering material are from the early months of the dry season, April-June, but first flowers likely bloom during the wet season (November-March) given the observation of mature fruits in April and onward. Under greenhouse conditions *S. jobsonii* fruits mature around 60 days after hand pollination. Successful *ex situ* autogamous and geitonogamous pollinations infer that the species is self-compatible.

##### Etymology.

The specific epithet of “jobsonii” is selected to honor Peter Jobson, Senior Botanist at the Northern Territory Herbarium at Alice Springs, an expert on the Northern Territory flora and the leader of the 2016 expedition to collect this and numerous other *Solanum* taxa with the authors.

##### Preliminary conservation status.

Cowie et al. (2011) noted that Limmen NP is home to nearly 1200 plant taxa, including two, *Seorsus intratropicus* (F.Muell.) Rye & Trudgen (Myrtaceae) and *Triodia longiloba* Lazarides (Poaceae), for which Limmen is considered the primary center of their distribution. *Solanum jobsonii* follows the pattern of these two taxa in its being restricted to specialized habitats and being largely known from this single national park, thus we suggest that it also be added to the park’s list of species of conservation significance (Cowie et al. 2011). The species is known from only four populations (even after much searching by the authors in other potentially-appropriate habitats), each consisting of a few dozen individuals. When evaluated using the IUCN Red List Categories and Criteria for extinction risk (IUCN 2001), *S. jobsonii* falls into the Vulnerable (VU) category under Criterion B (B1ab(iii)+2ab(iii)). The VU designation is the lowest of three threatened categories, but indicates the taxon still faces a high risk of extinction in the wild. It has an Area of Occupancy < 2000 km^2^ and Extent of Occurrence < 20,000 km^2^, less than 10 known locations that are possibly fragmented, and observed decline in overall habitat quality. Suitable habitat will continue to decline without active conservation management.

##### Specimens examined.

**AUSTRALIA. Northern Territory**: Limmen National Park, just north of Lorella Springs turn off, 15°54'44"S, 135°31'42"E, 17 April 2008 (fl, fr), *D.J. Dixon 1745* (DNA); Limmen National Park, Nathan River Road, 3.7 km north of Lorella Springs turnoff, 15.94913°S, 135.53464°E, elev. 246 ft., 14 May 2016 (fl, fr), *C.T. Martine, J.T. Cantley, L.M. Lacey and P. Jobson 4226* (DNA, BUPL); Limmen National Park, jct. Lorella Springs Rd. and Nathan River Rd., 15.91605°S, 135.52926°E, 14 May 2016 (fl, fr), *C.T. Martine, J.T. Cantley, L.M. Lacey and P. Jobson 4227* (DNA, BUPL); Benmara Station, approx. 1 km west No. 38 Bore, 17°54'--"S, 136°57'--"E, 5 June 1984 (fl), *Strong 253* (DNA); Limmen National Park, along main road, 16°01'39"S, 135°33'24"E, 8 May 2010 (fl, fr), *B. Stuckey & I.D. Cowie 595* (DNA); Savanna Way between Nathan River and Borroloola, 15°47'44"S, 135°25'46"E, 15 July 2008 (fl), *H. van der Werff & B. Gray 222501* (DNA); Limmen National Park P, Nathan River Rd., Lorella Springs turnoff, 15°54'56"S, 135°31'46"E, 12 May 2010 (fl, fr), *B. Wirff 531* (DNA).

##### Discussion.

Morphological comparisons of *S. jobsonii* and its close relatives, *S. watneyi*, *S. eburneum*, and *S. diversiflorum*, demonstrate a statistically significant difference among the four taxa. Most notably, *S. jobsonii* differs from the other three species by its involute corolla margins, deeply dissected leaves with 6-12 lobes, smaller creamy-yellow fruits, and a set of nine morphometric characters highlighted in Table 1[including corolla diameter (in both staminate and hermaphrodite flowers), calyx lobe length, and length of the fruiting pedicel]. While leaf lobing can be a rather variable character within and between *Solanum* species, the deeply-cut sinuses of *S. jobsonii* (and the occasionally linear leaf lobes/blades) are quite visually distinctive – and this character holds up in comparison to the close relatives in both field collections and in cultivation. The new species maintains a distinct geographic distribution from its closely related congeners, inhabiting limited areas on gray-brown clayey soil with limestone stones and laterite pebbles in the region of Limmen National Park in the northeastern portion of the Northern Territory. The species is named after Peter Jobson, Senior Botanist at the Northern Territory Herbarium at Alice Springs, who organized and led the expedition during which the new species was most recently collected and confirmed as distinct. *Solanum jobsonii* is one of the first new plant species described from Limmen NP, an area that received formal protection in only 2012.

National parks are under threat throughout the world, with federally-protected lands in places like the United States in potential danger of being left unfunded, deforested, or sold into private ownership (The Guardian 2017). By contrast, the 10,000 km^2^ Limmen NP is a new acquisition for the Northern Territory Government and, in its short time under protection, has already proved to be a cradle of impressive biodiversity (Cowie et al. 2011). Notably, the use of trained biodiversity scientists in surveys of the proposed parkland provided masses of data in support of protecting this area as a national treasure. The discovery of the new species described here, and the potential description of other new forms of biodiversity from Limmen NP, is a testament to the benefits of not only investing in national parks in Australia and elsewhere, but also investing in parks-based scientific inquiry.

## Supplementary Material

XML Treatment for Solanum jobsonii
